# Telehealth-Based Services During the COVID-19 Pandemic: A Systematic Review of Features and Challenges

**DOI:** 10.3389/fpubh.2021.711762

**Published:** 2021-07-19

**Authors:** Farnaz Khoshrounejad, Mahsa Hamednia, Ameneh Mehrjerd, Shima Pichaghsaz, Hossein Jamalirad, Mahdi Sargolzaei, Benyamin Hoseini, Shokoufeh Aalaei

**Affiliations:** ^1^Department of Medical Informatics, Faculty of Medicine, Mashhad University of Medical Sciences, Mashhad, Iran; ^2^Department of Computer Engineering, Ayatollah Amoli University, Science and Research Branch, Amol, Iran; ^3^Pharmaceutical Research Center, Mashhad University of Medical Sciences, Mashhad, Iran

**Keywords:** telehealth, telemedicine, COVID-19, coronavirus, SARS-CoV-2, pandemic, disaster management

## Abstract

**Background:** As an ever-growing popular service, telehealth catered for better access to high-quality healthcare services. It is more valuable and cost-effective, particularly in the middle of the current COVID-19 pandemic. Accordingly, this study aimed to systematically review the features and challenges of telehealth-based services developed to support COVID-19 patients and healthcare providers.

**Methods:** A comprehensive search was done for the English language and peer-reviewed articles published until November 2020 using PubMed and Scopus electronic databases. In this review paper, only studies focusing on the telehealth-based service to support COVID-19 patients and healthcare providers were included. The first author's name, publication year, country of the research, study objectives, outcomes, function type including screening, triage, prevention, diagnosis, treatment or follow-up, target population, media, communication type, guideline-based design, main findings, and challenges were extracted, classified, and tabulated.

**Results:** Of the 5,005 studies identified initially, 64 met the eligibility criteria. The studies came from 18 countries. Most of them were conducted in the United States and China. Phone calls, mobile applications, videoconferencing or video calls, emails, websites, text messages, mixed-reality, and teleradiology software were used as the media for communication. The majority of studies used a synchronous communication. The articles addressed the prevention, screening, triage, diagnosis, treatment, and follow-up aspects of COVID-19 which the most common purpose was the patients' follow-up (34/64, 53%). Thirteen group barriers were identified in the literature, which technology acceptance and user adoption, concerns about the adequacy and accuracy of subjective patient assessment, and technical issues were the most frequent ones.

**Conclusion:** This review revealed the usefulness of telehealth-based services during the COVID-19 outbreak and beyond. The features and challenges identified through the literature can be helpful for a better understanding of current telehealth approaches and pointed out the need for clear guidelines, scientific evidence, and innovative policies to implement successful telehealth projects.

## Background

The Coronavirus disease 2019 (COVID-19) pandemic started in Wuhan (China) in December 2019 and was spread worldwide (1). Thus the World Health Organization (WHO) described the disease as an epidemic (2). The number of cases affected by COVID-19 is rising rapidly. According to the latest WHO reports, there are over 163 million confirmed cases of COVID-19 with more than three million deaths (18 May 2021). Older people and those with a weak immune system are at a higher risk of contracting this virus; however, others are still susceptible (3, 4). The coronavirus spread mainly through person-to-person contact from respiratory droplets (5).

To reduce the transmission of the virus, several infection control strategies were developed such as “social distancing” and “self-isolation.” Relevant guidelines were created and the mobility of people was restricted and affected in their daily lives (6). Therefore, the critical conditions influenced by the widespread prevalence of COVID-19 led to significant changes in medicine, and the way physicians diagnose the disease and interact with patients has quickly changed, too (7). Several correlational studies showed that telehealth/telemedicine systems should be considered part of the response to the outbreak of COVID-19 by healthcare systems (8).

There are various definitions for telemedicine. According to WHO definition, “telemedicine is the delivery of healthcare services, where distance is a critical factor, by all healthcare professionals using information and communication technologies for the exchange of valid information for the diagnosis, treatment, and prevention of disease and injuries, research and evaluation, and for continuing education of healthcare providers, all in the interest of advancing the health of individuals and their communities” (9). According to the Centers for Medicare and Medicaid Services (CMS) (10), “telemedicine seeks to improve a patient's health by permitting two-way, real-time interactive communication between the patient and the physician at a distant site'. Sood et al. (11) found 104 peer-reviewed definitions of the word. They came to the conclusion that telemedicine is a subset of telehealth, and the two terms should not be used interchangeably due to some differences (12). Telehealth is an expansion of telemedicine. However, unlike telemedicine, which focuses solely on the curative aspect, it encompasses the field's preventative, promotive, and curative elements. Given the broad spectrum of teleservices available, from prevention to follow-up during the pandemic, we used the term “telehealth” to cover a broader set of activities.

Telehealth systems can significantly improve the triage, treatment, and care of patients, particularly where there is a restriction of the available resources (8). The use of telehealth is one of the services that should be offered by healthcare providers to continue patient care while minimizing the risk of exposure to or transmission of COVID-19 (13). In China, areas with poor access to healthcare services reported a higher case fatality rate of COVID-19 than those with sufficient access to healthcare services (14). Thus, telehealth might provide remote or virtual care services to patients with less access to face-to-face care.

Numerous studies have been conducted worldwide using telehealth in response to the COVID-19 pandemic in various fields such as dermatology (15), psychology (16), cancer (17), and so on. These studies were performed to provide healthcare to patients while reducing the transmission of COVID-19 to patients, families, and healthcare workers (18). Considering the capability of telehealth approaches, the widespread use of telehealth-based services is not far from expectation during the pandemic. The frequent related scientific articles and publications prove the importance of this issue.

As telehealth becomes more widespread, further research should be conducted to rigorously evaluate and address the properties and challenges of services to ensure this approach is used wisely and thoughtfully in response to COVID-19. To the best of our knowledge, so far, there has been little research as systematic reviews of telehealth-based studies to support COVID-19 patients and healthcare providers. So, there indeed remains the need to examine the different aspects of telehealth studies to have a better understanding of the current situation, challenges, and gaps of telehealth-based services to design more effective interventions. Accordingly, this review aimed to explore more specific properties of telehealth-based services, which were developed to support patients suspected of or afflicted with COVID-19 and healthcare providers. Moreover, this study helps plan future work that may eventually lead to higher quality information technology (IT) tools as a telehealth-based solution.

## Methods

This review follows the preferred reporting items for systematic reviews and meta-analyses (PRISMA) guideline (19) for identifying potentially related articles to COVID-19 telehealth.

### Data Sources and Search Strategy

A comprehensive search was done on the PubMed and Scopus electronic databases for articles published until 15 November 2020. We prepared the search terms using the PICO approach, which stands for patient, problem or population (P), issue of interest or intervention (I), comparison, control or comparator (C) and outcome (O). As the search aimed to be as comprehensive as possible and corresponding to the research questions, two concepts including COVID-19 for problem/population and telehealth for issue of interest /intervention were used to build the search strategy. All studies, regardless of the outcome and study design, could be included in the study. A combination of keywords and controlled vocabulary terms related to the target concepts was used. To search for a combination of terms, the Boolean operators (AND, OR, and NOT) were employed. Searching each database was consequently altered. For instance, the search strategy used in PubMed is presented in Table 1. The search strategy was conducted by two authors (SA and BH) independently and confirmed by all other members of the research team.

**Table 1:** Keywords and controlled vocabulary terms combination in the search strategy based on PICO approach.

**Concepts**	**Keywords combination**
Problem: Telehealth	telemedicine OR tele-medicine OR “Remote consultation” OR “Remote consultations” OR teleconsultation OR tele-consultation OR telehealth OR tele-health OR telerehabilitation OR tele-rehabilitation OR “remote rehabilitation” OR telepsychology OR teledermatology OR teleradiology OR Telepsychiatry OR “remote radiology” OR telehepatology OR “Home care services” OR Telenursing OR Telepractice OR “Tele-practice” OR “Remote Care” OR “Community medicine” OR tele-nurse OR tele-nursing OR “tele nursing” OR telecare OR tele-care OR Telehomecare OR “Tele home care” OR Tele-homecare OR “home monitoring” OR home-monitoring OR telecommunication OR telecommunications OR “tele rehabilitation” OR “tele rehabilitations” OR telecare OR “tele care” OR tele-care OR tele-home OR telehome OR “tele visit” OR e-health OR “e health” OR ehealth OR “remote assessment” OR “remote treatment” OR telemonitoring OR tele-monitoring OR “video consultation” OR “Video Consultations” OR “Remote Monitoring” OR “Remote Monitor” OR Telemetry OR mhealth OR “mobile health” OR “Digital Health” OR “smart phone” OR “Cellular phone” OR “Cell phone” OR “mobile app” OR “Remote Sensing Technology” OR “Remote Sensing Technologies” OR Videoconference OR Videoconference OR “Video Conference” OR “Video Conferencing” OR “Video consultation” OR “Video Consultations” OR “Video Visit” OR “Video Visits” OR “Video Call” OR Robot OR TeleCheck
Issue of interest: Corona	“coronavirus disease-2019” [ti] OR “coronavirus disease 2019” [ti] OR “Wuhan seafood market pneumonia virus” [ti] OR “Severe acute respiratory syndrome coronavirus 2” [ti] OR Covid-19 [ti] OR SARS-CoV-2 [ti] OR 2019-nCoV [ti] OR “Wuhan coronavirus” [ti] OR “2019 novel coronavirus” [ti]

### Study Selection

#### Inclusion Criteria

Studies were deemed eligible to be included in the review if they:

1. Used telehealth tools for the screening, triage, prevention, diagnosis, treatment, or follow-up of COVID-19 patients suspected of or afflicted with COVID-19.
2. Used telehealth tools to reduce the healthcare providers' exposure to COVID-19.
3. Were published in scientific journals
4. Were published until 15 November 2020.

#### Exclusion Criteria

Studies were excluded if they:

1. Were not available in full text.
2. Were not in the English language.
3. Were reviews, conference proceedings, opinion articles, letters to editorial, commentaries, and viewpoint. Also, since the purpose of this study is to provide an overview of the services offered in a population and also examine challenges of using services in communities, studies that report results of using telehealth for one person were excluded. This is because the usefulness of service to one person cannot be generalized to a population.
4. 4Used telehealth to facilitate service provision during the COVID-19 pandemic for patients with other diseases (than COVID-19).
5. Used telehealth to provide irrelevant services to the coronavirus.
6. Focused only on assessing participants' attitude toward telehealth services without any results of the deployment of the telehealth services.
7. Used telehealth tools to collect data from patients to test some research hypothesis with no immediate and direct benefit for patients or healthcare providers.
8. Of the articles published on the same tool, only the report with more complete results was included.

### Screening and Eliminating Irrelevant Sources

First, duplicate articles were eliminated from the retrieved articles. Then, four of the authors independently screened articles based on titles and abstracts to identify the studies that potentially could fit into the research question and meet the eligibility criteria. A record would be excluded if it was marked irrelevant by at least three of the reviewers. If it was difficult to decide based on the title or abstract, the full text was scrutinized. When a consensus was lacking, two senior researchers in the study (SA and MS) were consulted for the final decision.

### Form Development and Data Extraction

A particular form was developed for data extraction. General and technical information were extracted from the included studies. For each included study, the first author's name, publication year, country of the research, study objectives, outcomes, function type, target population, media, communication type, guideline-based design, main findings, and challenges were extracted.

The outcomes were classified based on a taxonomy developed by Dood et al. which is used to classify outcomes included in all trials, core outcome sets (COS), systematic reviews, and trial registries (20). It has five core area: death, physiological/ clinical, life impact, resource use, and adverse events.

Regarding the function type, we considered six categories including screening, triage, prevention, diagnosis, treatment and follow-up, which defined as below:

**Screening:** using tools by symptomatic and asymptomatic cases to detect COVID-19 suspected or confirmed patients.

**Triage:** using tools by symptomatic patients or providers to guide them about the necessary action to do based on the severity of symptoms (deterioration of patients health status).

**Prevention:** using tools by patients (symptomatic and asymptomatic cases) to prevent contamination of healthy people and also using tools by providers to protect them during visiting COVID-19 suspected or confirmed patients.

**Diagnosis:** using tools to help patients or providers to do diagnosis procedures accurately and adequately.

**Treatment:** using tools to provide recommendations to and decrease symptoms in symptomatic patients (almost confirmed cases).

**Follow-up:** using tools to monitor the health status of inpatients discharged from the hospital or outpatients who were recommended staying at home.

Regarding the challenges, for emerging telehealth approaches considering COVID-19 complications, it is essential to acknowledge their barriers and challenges. To highlight and better understand these challenges and significant barriers identified through the included literature, we have extracted and categorized them based on the topic. So similar topics were placed in a category based on consensus between research team members and each category was named according to its theme.

The data were extracted by FK, MH, SP, and AM and finally revised and confirmed by SA and MS to ensure the accuracy of the extracted data.

### Data Synthesis and Analysis

The data analysis began with a summary of the study and the properties of the telehealth systems, along with the extracted data tabulated. The data were categorized at the same time as tabulating them. The aim was to manage a range of values for each variable to take. The reviewers recurrently refined the categories by introducing new categories and letting older versions be omitted or merged.

A narrative synthesis was done for the expression of results that were reported in the studies. This was done by comparing and contrasting the data. The data that was obtained from the studies were qualitatively elaborated and presented. To solve any case of disagreement, the authors met several times until a consensus was reached.

### Quality Assessment

The present review did not aim to assess the effect of the reviewed systems or the quality of the target studies. Nor did it strive to make any conclusion or generalization based on the literature reviewed. We sought to identify the properties of telehealth systems and recognize the state of these systems at the time of the COVID-19 pandemic. Accordingly, the quality of the content of the included studies was not assessed.

## Results

**Figure 1:**
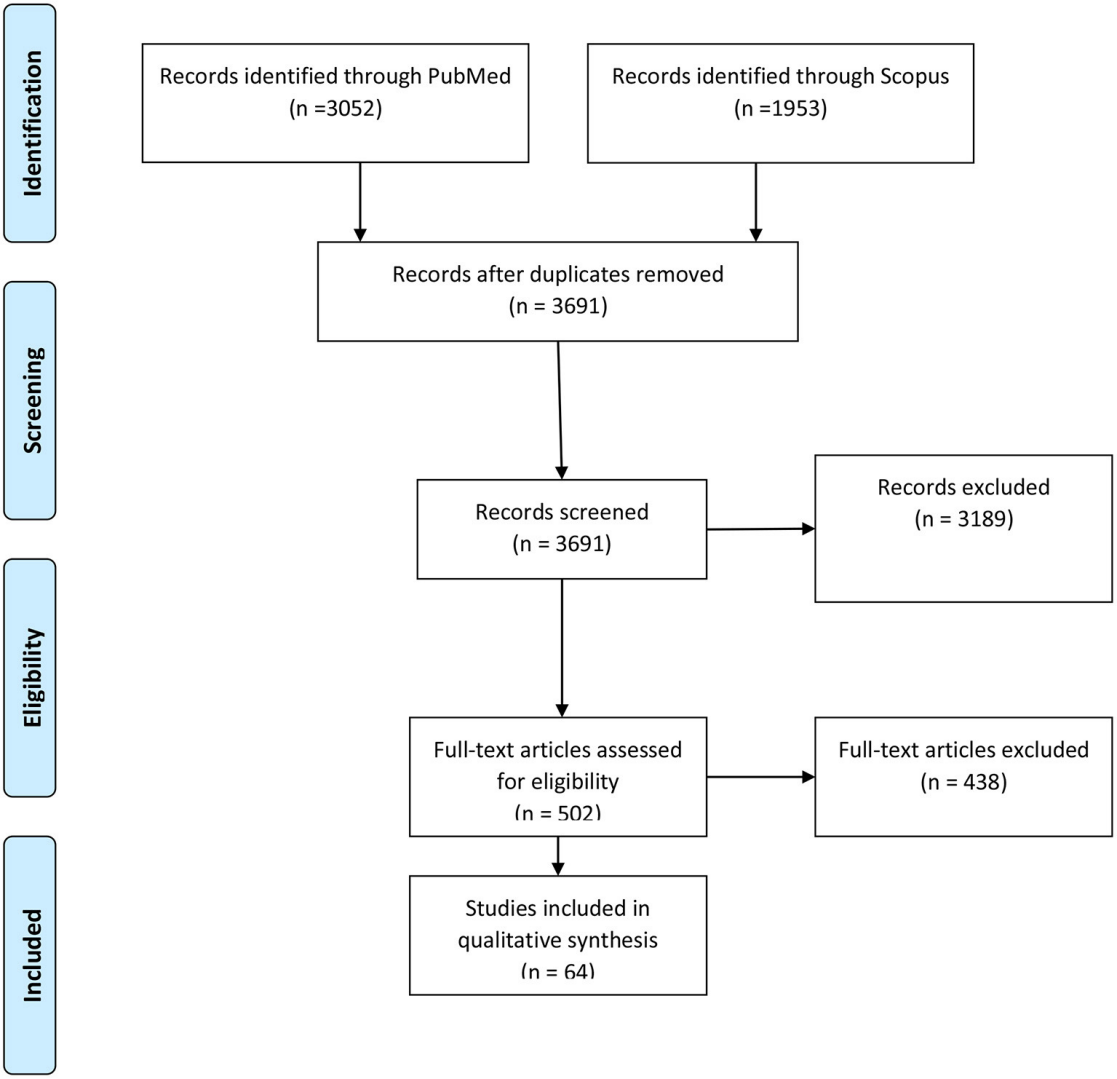
The PRISMA flow diagram of the included studies in the review.

The PRISMA diagram of the study selection process is shown in Figure 1. Searching the two databases resulted in 5,005 records, among which 3,691 papers were screened by title or abstract after removing the duplicates. Three thousand one hundred and eighty nine studies were excluded because of apparent irrelevance in the first step or using non-English languages, or the lack of full text. Finally, the full texts of 502 articles were assessed for eligibility, while 64 met all the eligibility criteria and were included for the stage of evidence synthesis.

### Overview of the Properties of the Included Studies

**Figure 2:**
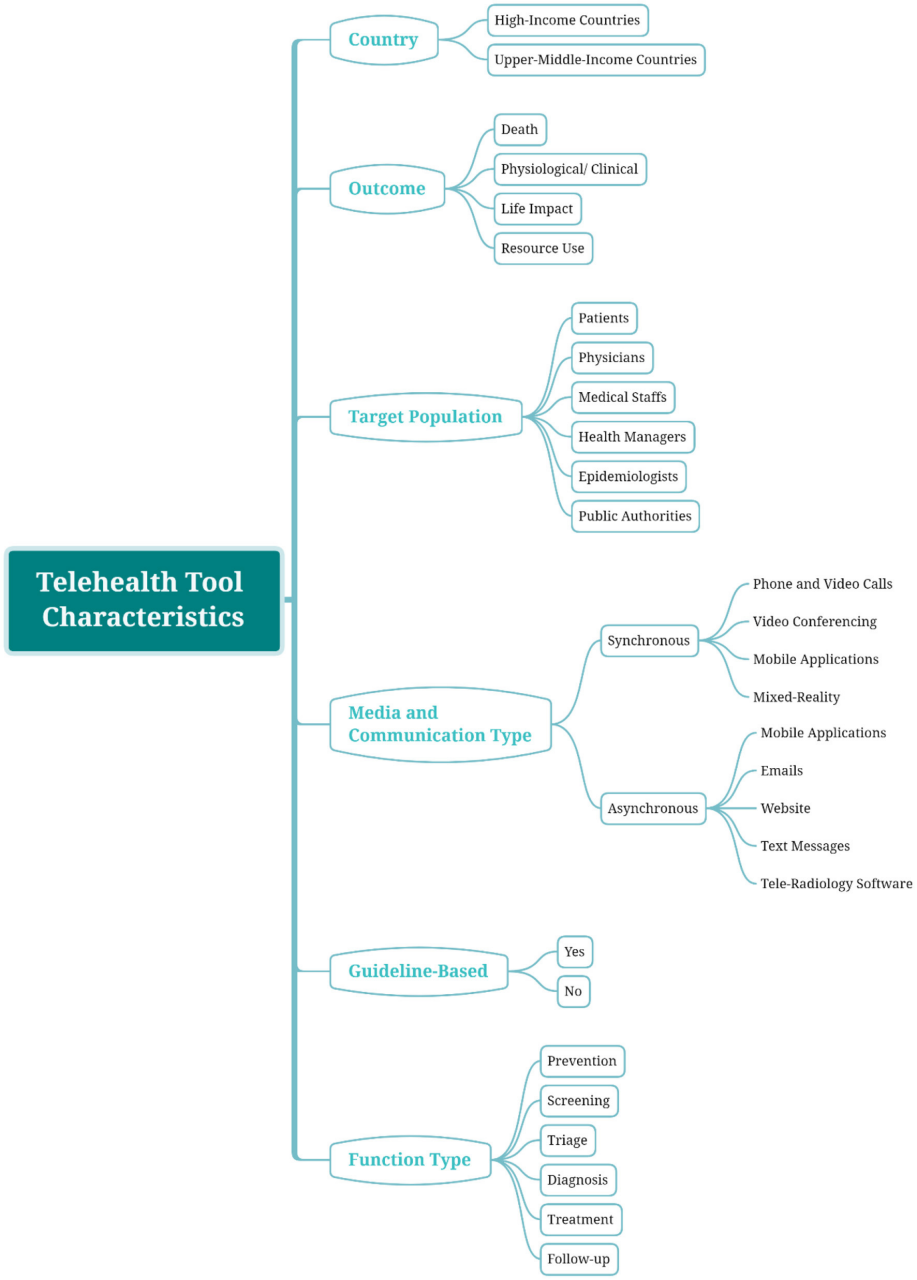
Classification map; an overview of the identified categories in each aspect.

Supplementary Table 1 presents the properties of the included studies. Also, all the findings through the reviewed paper are summarized in Figure 2.

### Date and Countries

**Figure 3:**
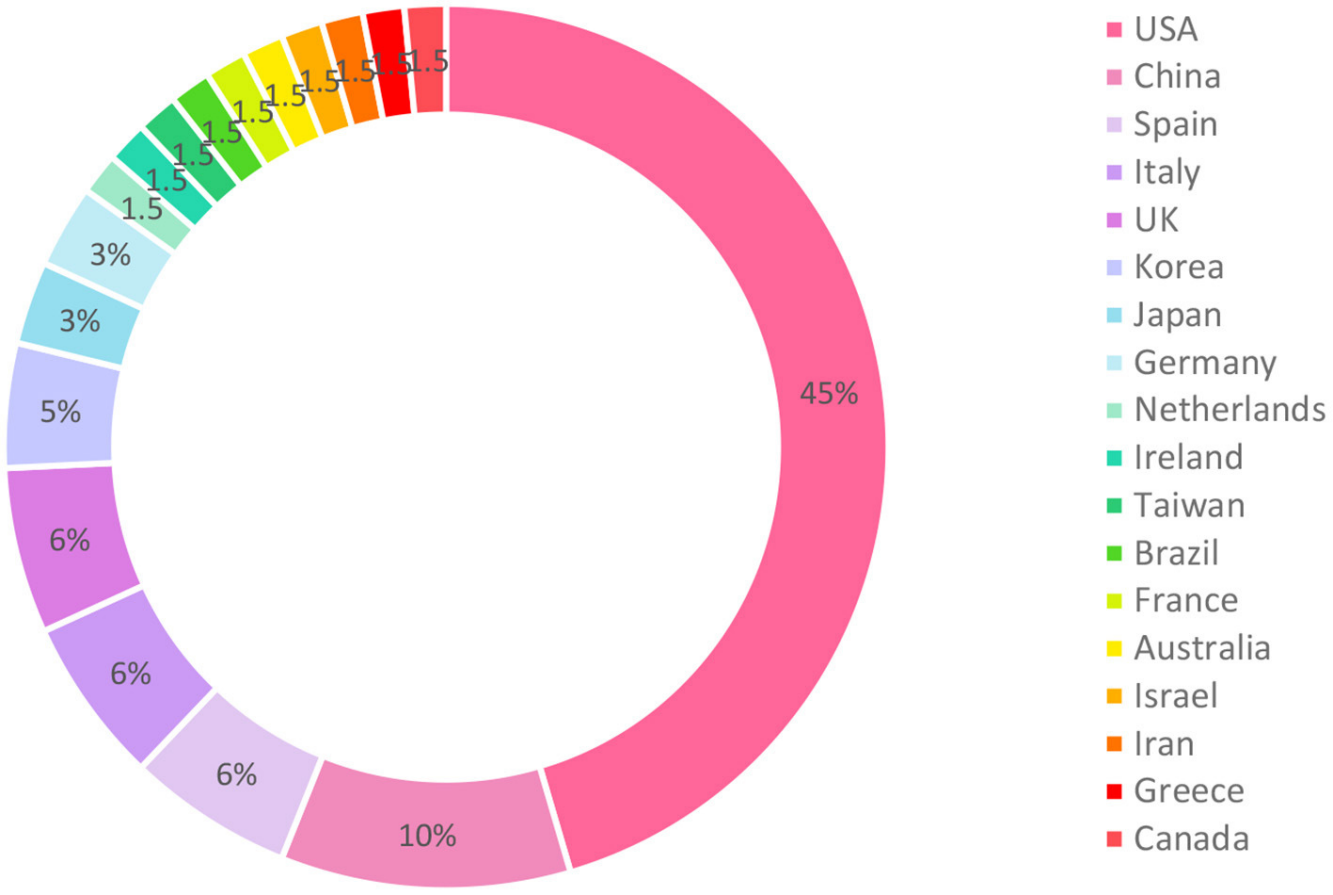
Distribution of included studies in the countries.

All of the included studies were published in 2020. The studies came from 18 countries. Most of them (30, 45%) were conducted in the United States and 7 (11%) were implemented in China. Four studies were conducted in Spain, four in Italy, four in the United Kingdom, three in Korea, two in Germany, and two in Japan. From Canada, Greece, Iran, Israel (joint to the United States), Australia, France, Brazil, Taiwan, Ireland, and Netherlands, only one article was included. One study was implemented jointly in the United States and the United Kingdom. Therefore, we reported the countries of that study separately. The vast majority of articles were published from high-income countries (*n* = 55) and the remaining from upper-middle–income countries. Figure 3 illustrated the distribution of included studies in the countries.

### Outcomes and Main Findings

In a significant number of the studies (27/64, 42%), a descriptive report was used to demonstrate system features, development process and patients' characteristics. The remaining outcomes classified into four categories, including death, physiological/clinical, life impact and resource use. The result of outcomes classification and frequency of each outcome is shown in Table 2. Overall, based on the studies' main findings, most studies reported improvement in outcome measures. Supplementary Table 2 showed the outcomes and main findings of the included studies.

**Table 2:** Outcomes classification and frequency.

**Core area**	**Outcome**	**Frequency**
Death	Mortality	2
Physiological/Clinical	Mental health status	1
	Psychological distress	1
	Glycemic outcomes	1
Life impact	System usage	6
	User's satisfaction	5
	Exposure time	3
	Feasibility reports	2
	Triage/Diagnostic accuracy	2
	Waiting time	2
	Mobility score	1
	Visit duration	1
	Response rate	1
	No show rate	1
	Time to visit scheduling	1
	Hospitalization rate	1
	Visit volume	1
	Biological sufficiency	1
	Measurement accuracy	1
	System effectiveness	1
	Patient's evaluation time	1
	Length of stay	1
	ICU admission	1
Resource use	PPE use	4

### Target Population

In four studies, the patients were the only ones involved in using telehealth services. The purpose of two of these studies was screening which mobile applications and mobile sensors via smartwatches were used to achieve the goal. Two remaining studies used mobile applications to prevent spreading coronavirus using contact tracking and treat psychological distress in COVID-19 patients.

In five studies, the physicians and hospital workers were the primary recipients of telehealth services. In two of these studies, teleconsultation services were established to connect physicians. One used tele radiography software to diagnose COVID-19 based on chest CT and the other used phone calls to share decisions between experts to manage children with or exposed to COVID-19 infection. In two studies, mobile applications were used for screening purposes to monitor and assess COVID-19 infections and compatible symptoms in health care workers. In one study, mobile application and mixed-reality headsets were used to protect health care providers during patients' visits.

In six studies, in addition to patients and physicians, health managers, epidemiologists, and public authorities were targeted to use the collected data for monitoring activities, public health planning, and managing large numbers of COVID-19 patients as well as overwhelmed hospital staff. In the remaining studies, a team including attending physicians, physician assistants, residents, nurse practitioners (NPs), registered nurses (RNs), social workers, alcohol and drug counselors, and office staff were involved in patients' care.

### Media and Communication Type

The extracted information indicates that 32 (50%) of the reviewed papers used synchronous communication, 11 (17%) of them used asynchronous communication, and 21 (33%) used both means of communication. Synchronous communication deals with the mechanisms of providing real-time healthcare services. In included studies, phone and video calls, videoconferencing, mobile applications and mixed-reality were used to establish real-time communications. In the asynchronous approach, also known as the store and forward mechanism, sending and receiving the data do not occur at the same time and the data are stored somewhere in the midway temporarily. Mobile applications, emails, websites, text messages and teleradiology software used for this type of communication.

Overall, phone calls (48%), mobile applications (45%), video calls and videoconferencing (42%), and email (12%) were the primary means of the needed communication.

### Guideline-Based Design

This variable indicates whether the telehealth tool was developed based on guideline recommendations or experts' collaboration or not. Supplementary Table 1 shows that guidelines and experts' collaborations had been used in 41 studies (64%) in tool development.

### Function Type

The function types of the telehealth services in the included studies were extracted and classified into six categories, which screening, triage, prevention, diagnosis, treatment, and follow-up. Out of the 64 included studies, the most common purpose (34/64, 53%) was the patients' follow-up. Twenty-two studies (34%) focused on the treatment, 21 focused on screening (33%), 14 (22%) involved the triage, nine (14%) aimed at prevention, and four (6%) focused on diagnosing COVID-19. Almost more than half of the studies (*n* = 34, 53%) reported using the service for a single function.

### Barriers and Challenges

Sixty-seven barriers were identified in 28 studies, categorized into the 13 groups as below:

1. Adequacy and accuracy of subjective patient assessment/accuracy of tele-tools
  - Providers' ability to undertake a comprehensive physical examination and measure vital signs may be restricted by this barrier. Moreover, the inaccuracy of tele-tools may compromise physicians' reliance on measurements.

2. Change in physician-patient communication
  - Healthcare providers that use telehealth services do not have direct contact with patients. This could have negative consequences and may have an adverse effect on the quality of physician-patient relationship, which is the foundation of clinical care.

3. Technology acceptance/user adoption
  - This barrier indicates patients' and physicians' reluctance to use telehealth services. This may be due to various reasons such as workload, lack of time, lack of workflow integration, users' lack of technical skills and some physical and cognitive impairment.

4. Data privacy and security
  - This barrier indicates concerns in case of safety, privacy, security, ownership, storage and traceability of personal health data for telehealth services which are often established on online platforms.

5. System design
  - This barrier points to the fact that specific features in the design of systems may reduce the widespread use of the systems. For example, manual data entry, daily data exports, considerable input and oversight and lack of real-time feedback to the user can affect adherence and system usage.

6. Resource availability/accessibility
  - Establishing telehealth needs robust technology infrastructure such as digital devices, smartphones, tablets, Wi-Fi connections and monitoring equipment, and human resources such as certified providers and technical staff.

7. Technical issues
  - Software functionality, slow processing speed, limited battery life and limited bandwidth are among technical problems. A poor internet connection due to non-broadband or low-speed broadband internet can cause dropped calls, delays, and poor quality audio and video. It can interrupt care delivery and lead to physicians' and patients' dissatisfaction with telehealth.

8. Standards and legal considerations
  - Several legal facets are involved in the implementation of telehealth that should be followed at the facility, state, and federal levels. Due to the constraints during the pandemic, it is challenging to meet the standards and regulations for telehealth.

9. Insurance policies and reimbursement
  - Lack of a clear reimbursement plan for telemedicine services provided by physicians can reduce their participation. Moreover, coverage and payment deficiency virtual services by patients' insurance can make patients prefer face-to-face communications with physicians.

10. Data availability/accessibility
  - Sufficient data is one of the most essential elements in patient care. Due to the nature of telehealth systems, collecting comprehensive data from patients may be impossible and consequently resulting in data unavailability. In some cases, despite the data availability, lack of interoperability between systems can limit data accessibility. For example if electronic health records (EHR) system does not coordinate with the telehealth platform, data cannot be accessible through routine workflow.

11. System maintenance
  - The volatile changing landscape of the COVID-19 pandemic leads to daily guidelines and protocol updates, which presents challenges to telehealth systems maintenance.

12. Presence of parallel systems
  - The design and development of various digital health tools in response to the ongoing COVID-19 pandemic can affect participant engagement in system use.

13. Different operational requirements in organizations and lack of widespread use
  - System development to meet local organizations' requirements limits its widespread use. This imposes a high cost on other organizations to redesign the system.

**Figure 4:**
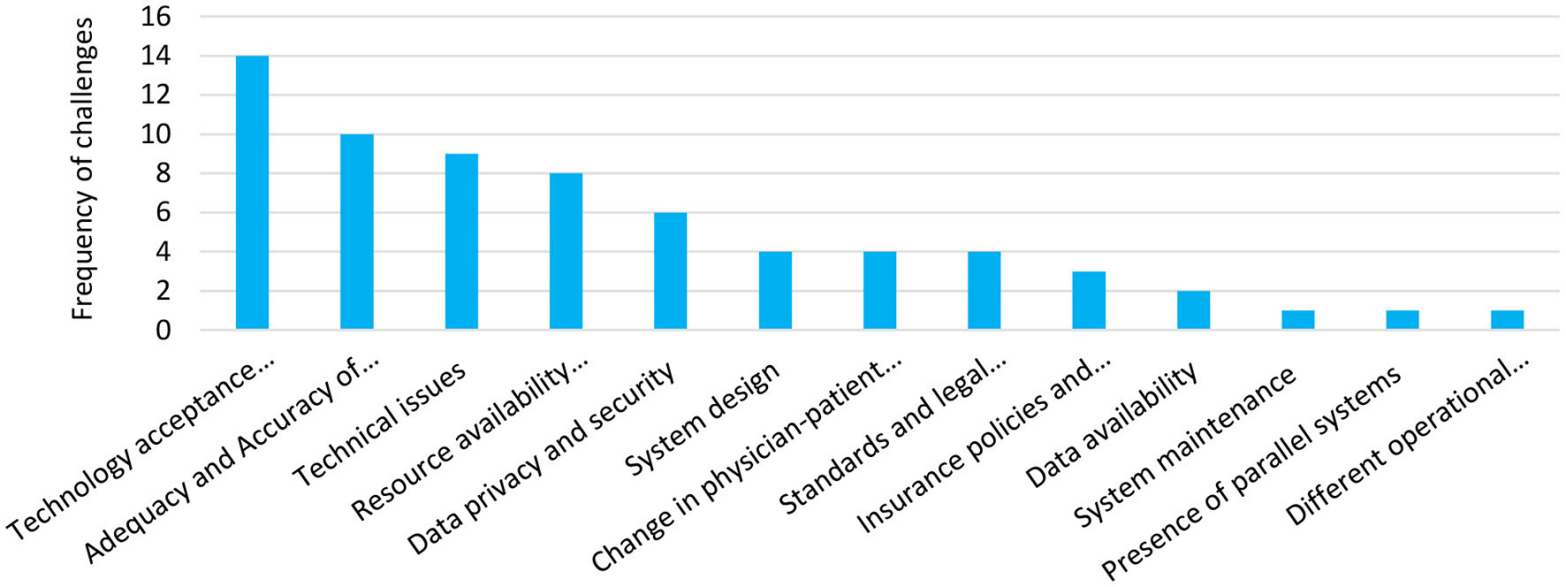
Identified barriers and challenges of telehealth establishment during COVID-19 pandemic and their frequency in included studies.

The frequency of each group is presented in Figure 4. Technology acceptance and user adoption, concerns about the adequacy and accuracy of subjective patient assessment, and technical issues were the most frequent.

## Discussion

This study aimed at providing an overview of features and challenges of COVID-19 telehealth solutions reported in published studies during the pandemic. The widespread use of telemedicine approaches in COVID-19 management, from screening to follow-up, shows the community's acceptance and interest in telehealth solutions. In the following, we will discuss the features and challenges of the telehealth solutions used in the studies.

### Target Population

The review of the research findings revealed the usefulness of the telehealth-based services during the COVID-19 pandemic for all stakeholders, including the general population, afflicted patients, healthcare providers, health managers, epidemiologists, and public authorities. This finding is consistent with many previous studies in this field (21–23). Supposedly, the reason lies that patients, healthcare providers, and policymakers were convinced that quarantine, minimum social attendance, and keeping a social distance are the leading solutions to reduce the transmission of the disease. In these circumstances, the best way to deliver healthcare services is through the utilization of telehealth capabilities.

### Guideline-Based Design

The use of telehealth technologies was dramatically accelerated as an effective, safe, and scalable way to help patients and healthcare providers during the pandemic. This might lead to a consensus to create new incentives, make helpful policies, and remove old barriers to the acceptance of telehealth usage. Indeed, the need for following guidelines and scientific evidence in implementing a telehealth system plays a crucial role in standardizing service provision for different patients. Besides, it helps to maximize compatibility with face-to-face healthcare services. Among the articles reviewed in the present study, 64% mentioned using guidelines or experts' collaborations in the telehealth tool development and service provision. Other studies (36%) have not noted using guidelines, and this is a matter of concern. Though during a pandemic, such an important matter can be easily neglected due to the existing crises, it is noteworthy that disobeying guidelines and standards can harm patients and make conditions even harder than the traditional forms of service provision.

### Function Type

Since the coronavirus has burdened societies and healthcare providers, many attempts are made to prevent more affliction. Moreover, due to the lack of clinical sources, the crowd of patients, and the limited capacity of hospitals, practical efforts are made to provide virtual care services for people. Thus, telehealth services have been used in different stages of healthcare including, prevention, screening, triage, diagnosis, treatment and follow-up. Due to the fast rate of the disease spread and its contagion, the number of potential patients afflicted with COVID-19 is high. Therefore, telehealth can be a complementary method to screen the patients and provide medical recommendations virtually (24, 25). A vast majority of articles reviewed in this study dealt with the follow-up (53%), treatment (34%), screening (33%), and triage (22%) purposes, respectively. Studies are listed by function types in Table 3.

**Table 3:** Function types in included studies.

**Function type**	**References**
Prevention	(26–36)
Screening	(13, 28, 30, 37–54)
Triage	(13, 29, 31, 32, 43, 45, 50, 55–61) )
Diagnosis	(7, 42, 60, 62)
Treatment	(13, 36, 39, 44, 45, 50, 52, 57, 58, 60, 63–73)
Follow-up	(13, 26, 28, 30, 39, 41, 42, 44, 50–52, 55, 57–59, 61, 63, 67, 68, 70–84)

Telehealth solutions that aimed at virtual triage and screening of patients can reduce the number of patients referred to medical centers; who their health status is not critical and didn't need in-person medical care. In this case, tele triage (13, 29, 31, 32, 43, 45, 50, 55–61) and tele screening (13, 28, 30, 37–43, 45–54, 85) can reduce the burden of overwhelmed hospital and healthcare providers.

Most of the telehealth services with preventive purpose (26–36) were used in emergency departments to protect physicians during visiting COVID-19 suspected or confirmed patients. Moreover, several symptoms and contact tracking applications were developed to prevent contamination of healthy people.

Regarding diagnosis purpose, in some studies, teleconsultation services were used by physicians to do diagnosis procedures. In some other studies, an attempt was made to perform the diagnosis process with the patients' participation accurately (7, 42, 60, 62).

In telehealth solutions with treatment purpose, the aim was to provide necessary recommendations to symptomatic patients to reduce their symptoms. Most of the patients who received this type of services were confirmed cases (13, 36, 39, 44, 45, 50, 52, 57, 58, 60, 63–73).

On the other hand, COVID-19 often affects a patient's health for a long time. So the patients need to be monitored regularly for their health status. Inpatients or outpatients with the stable condition are recommended to continue treatment and recovery at home due to the lack of hospital capacity. Telehealth systems with follow-up purposes aimed at keep tracking of patients who still require care (13, 26, 28, 30, 39, 41, 42, 44, 50–52, 55, 57–59, 61, 63, 67, 68, 70–84).

Due to the fast rate of the disease spread and its contagion, the number of potential patients afflicted with COVID-19 is high. Therefore, telehealth can be a complementary method to screen the patients and provide medical recommendations virtually (24).

### Media and Communication Type

There are two fundamental approaches to transfer data for telehealth: synchronous and asynchronous. The former deals with the mechanisms of providing real-time healthcare services. In the asynchronous approach, the data are stored at some points between the sender and recipient, i.e., sending and receiving the data do not occur simultaneously. In the reviewed papers, both synchronous and asynchronous approaches were used for communication. The former was the most prevalently used approach in which, audio and video calls, mobile applications, and mixed-reality were used for communication through a phone or other devices. It seems that making synchronous audio and video communication was most welcomed by patients and healthcare providers. The quality, availability, and patients and providers satisfaction of healthcare services would be enhanced through real-time communication. However, its cost-effectiveness is still open to controversy. Making such communication requires a reliable infrastructure to transfer data, especially voices and videos. Lacking a dedicated infrastructure and technical issues can result in a poor transmission discussed later in the challenges subsection. In asynchronous communication, the most prevalent tool was mobile applications.

### Countries

In the body of research reviewed, the most frequent relevant studies to the research question, respectively, belonged to United States (28/64) and China (7/64). In the United States, facilitating reimbursements for telehealth services by insurance companies and, consequently, the recent rapid increase in telehealth in that country can be one of the reasons for this number of articles (21). On the other hand, China was the first country engaged with the disease and spent many sources on this issue. So, it has applied a significant number of tele tools to fight the pandemic (86). Now the question is raised: Why are there a few studies conducted in China and other countries compared to the United States on the topic of telehealth in COVID-19? Note that most investigations conducted globally have been experimental in type and few studies have been done in the real environment and a large population. Because of the critical conditions of the pandemic, there has been no time for trial and error. Therefore, policymakers' emphasis was on the telehealth plans with a high probability of effectiveness. Moreover, many of these projects might have been left unreported as an article in scientific journals despite being effective. They may have been reported in the news and other resources (86). Thus, the number of publications in a country related to COVID-19 telehealth cannot necessarily be a reliable criterion to estimate the volume of activities in countries with this concern.

### Outcomes

Investigating reviewed papers revealed that a significant number of the studies (42%) only described system features, development process and patients' characteristics and didn't evaluate the effect of telehealth solutions on health, economic and feasibility related outcomes. The existing body of research on the mere development and deployment of a telehealth-based solution without any report of the appropriate outcomes can hardly contribute to expanding the telehealth domain during the pandemic. They scarcely provide a reasonable criterion for decision-making on the employment or unemployment of telehealth by policymakers. In the studies that have reported objective outcomes, the results show improvement in outcome measures. This shows the usefulness of telehealth solutions in pandemic management.

### Barriers and Challenges

In the body of research reviewed here, various factors were mentioned as barriers to the deployment of telehealth services. Technology acceptance and user adoption was the most common barriers against using telehealth solutions (13, 26, 28, 32, 34, 35, 42, 43, 50, 53, 61, 78, 83, 87). Several reasons were raised by physicians and patients for not willing to use new telehealth tools, including lack of time, lack of workflow integration, workload, difficulties with technology, lower levels of internet use, lack of confidence with technology, sensory impairments, health literacy, hearing and vision impairment, and so on. As pinpointed by Anthony Jnr. Bokolo (88), organizational, technological, and social factors play an essential role in accepting telehealth. Considering the role of physicians as key people in providing health services and using IT tools to serve telehealth purposes, it is essential to take these factors into account (88). Therefore, adequate instructions should be provided in the simplest and most accessible way for physicians to admit using IT tools for the telehealth establishment (18, 89). Moreover, considering the workload of physicians, especially during the pandemic, and the natural resistance of individuals against changes, telehealth should be integrated into their clinical workflow to impose the least burden (89). The fast spread of the disease has led to particular tele-based strategies to protect the hospital staff. Although some of these strategies were against the current workflow, they were well-accepted by the medical staff due to the present critical conditions. For instance, providing appropriate protection for the emergency department (ED) staff and minimizing the use of personal protective equipment (PPE), using onsite telemedicine increase safety for both physicians and nurses (27). Adequacy and accuracy of subjective patient assessment and accuracy of tele-tools were identified as the second barrier (24, 28, 35, 40, 43, 49, 61, 64, 82, 83). This issue was raised by physicians because they believe tele visit limits the ability of providers to perform a complete physical examination and measure vital signs. They also claim that tele visit leads to change in physician-patient communication which is a foundation of clinical care (33, 35, 64, 65). Technical issues due to lack of dedicated IT infrastructure was another barrier (26, 27, 33, 42, 50, 61, 64, 73, 77). This challenge particularly affects synchronous service delivery. A poor internet connection can lead to poor quality audio and video. Forcing people to find alternative approaches might lead to dissatisfaction with telehealth services. For instance, Chou et al. (27) reported a technical issue of connectivity and Wi-Fi signal. The signal was not stable enough for videoconferences in some areas of the emergency department, so they had to use either the audio function or the intercom instead. Due to this problem, several physicians and nurses initially preferred phone interviews in that research. Different operational requirements in organizations can limit the widespread use of technology (54). Data availability (64, 73), resource availability including equipment and human resources and their accessibility for patients and providers (26, 28, 34, 36, 38, 61, 73, 75), standards and legal considerations (13, 27, 42, 50), insurance policies and reimbursement (26, 61, 65) and data privacy and security (13, 26, 27, 34, 35, 53) are among the barriers to using telehealth. However, some studies have overcome some barriers by presenting novel solutions (30, 35). System design and lack of necessary features embedded in telehealth systems (26, 28, 53, 75), system maintenance due to daily guideline and protocol updates (50), and presence of parallel systems (53) are challenges which needs a participatory design approach with the collaboration of various clinical and technical groups and policymakers in systems design.

### Limitation

However, our study had some limitations to be addressed. First, we searched only two databases, including PubMed and Scopus. This may affect the search comprehensiveness. Although, ~80–90% of studies conducted in telemedicine were accessible on PubMed (90), but as the search aimed to be as comprehensive as possible Scopus database was also searched. Second, limiting the search strategy to English-language studies may introduce a language bias. However, the English language is generally perceived as the universal language of science and studies highlighted. Overall, there is no evidence for a systematic bias from English language restrictions in systematic reviews in medical sciences (91–93). Third, as COVID-19 is growing at an unprecedented rate, the scientific community has attempted to provide its evidence-based findings to the public as soon as possible. So that this information can help slow down the spread of the disease and even manage to stop it. Thus, many of the papers published during the COVID-19 pandemic did not follow a standard format, and they mostly lacked factual data that can be drawn directly from the article. So to solve the variation in data extraction, we used several independent reviewers and all extracted data were double-checked and verified by two senior reviewers. Finally, considering the existing variety in the body of research on COVID-19 and the importance of the issue, in order not to miss any study, the present review took into account all papers published about telehealth during the coronavirus pandemic from different aspects. Though this inevitably reduced the sensitivity of the search, the authors are to a great extent sure that they did not miss any relevant study in line with the purpose of the review.

## Conclusion

Considering the capability of telehealth approaches, the widespread use of their services is not far from expectation during the pandemic. Telehealth solutions can provide services in pandemics in terms of prevention, screening, triage, diagnosis, treatment and follow-up. The identified features and barriers of telehealth tools through the reviewed papers can be helpful for a better understanding of current telehealth approaches in response to COVID-19. The identified barriers point out the need for clear guidelines, scientific evidence, and innovative policies to implement successful telehealth projects.

## Data Availability Statement

The original contributions presented in the study are included in the article/supplementary material, further inquiries can be directed to the corresponding author/s.

## Author Contributions

SA is the principal investigator of the study. SA, FK, and BH were responsible for the concept/idea/research design. Study selection was conducted by FK, MH, SP, AM, and HJ. Data extraction was done by FK, MH, AM, SP, and HJ. SA, FK, and MS drafted the manuscript. All authors read and approved the manuscript.

## Conflict of Interest

The authors declare that the research was conducted in the absence of any commercial or financial relationships that could be construed as a potential conflict of interest.

## Abbreviations

COVID-19: Coronavirus disease 2019
WHO: World health organization
ED: emergency department
PPE: personal protective equipment
IT: information technology.
