# Supplementary material for: Telehealth-Based Services During the COVID-19 Pandemic: A Systematic Review of Features and Challenges
Source: Front Public Health. 2021 Jul 19;9:711762. doi: 10.3389/fpubh.2021.711762 (PMC8326459; doi:10.3389/fpubh.2021.711762)
Supplement: Supplementary file 1 [file Table_1.docx]

**Tables**

**Supplementary Table 1: Properties of the included studies**

| **First author, Date** | **Country** | **Income level** | **Study objectives** | **Function type** | **Target population** | **Communication type** | **Media** | **Guideline-based** |
| --- | --- | --- | --- | --- | --- | --- | --- | --- |
| Heo et al. (2020) | Korea | High income | Developing a web-based application based on an algorithm and a series of simple questions that helps patients decide when to seek medical care | Screening | Patients | Synchronous | Mobile application | Yes |
| Lam et al. (2020) | Canada | High income | Developing and testing the feasibility of a virtual care program for self-isolating outpatients diagnosed with COVID-19 | Follow-up | Patients, physician | Both | Phone call, videoconference | Yes |
| Kouroubali et al. (2020) | Greece | High income | Describing the design of a platform, dynamically adapted according to patient preferences and medical history, to support patient-centered information, management and reporting of symptoms related to COVID-19 | Treatment, follow-up | Patients, physician, public authorities | Both | Mobile/desktop application | Yes |
| Jethwa et al. (2020) | USA | High income | Developing a COVID-19 virtual clinic (CVC) to screen and follow-up patients | Screening, follow-up | Patients, physician, clinical team (nurse, …) | Synchronous | Phone call, videoconference | Yes |
| Ford et al. (2020) | USA | High income | Describing the implementation and initial utilization of COVID-19 telehealth programs and 4 biomedical informatics innovations to screen and care for COVID-19 patients. | Screening, treatment, follow-up | Patients, physician | Both | Mobile application, phone call, video call | Yes |
| Krenitsky et al. (2020) | USA | High income | Designing and implementing a virtual telemonitoring COVID-19 clinic to close tracking of pregnant and postpartum women via the integration of telehealth visits, remote patient monitoring devices, and electronic medical record keeping. | Follow-up | Patients, physician, office staff | Synchronous | Mobile application, phone call, video call | No |
| Heslin et al. (2020) | USA | High income | Designing and implementing a telehealth program to establish two way communication between patients and providers in ED to decrease exposure to COVID-19 and conserving PPE | Prevention, triage | Patients, physician, clinical team (nurse, …) | Synchronous | Mobile/desktop application, video call, videoconference | No |
| Kassaye et al. (2020) | USA | High income | Develop a monitoring and reporting system for COVID-19 to support institutions conducting monitoring activities without compromising privacy | Screening, prevention, follow-up | Patients, institutions and agencies | Asynchronous | Website | Yes |
| Judson et al. (2020) | USA | High income | Deploy a digital patient-facing self-triage and self-scheduling tool | Triage | Patients, physician, clinical team (nurse, …) | Both | Phone call, video visit, patient portal messages | Yes |
| Harris et al. (2020) | USA | High income | Design and implementation of telehealth-centered strategies to improve outcomes in facility outbreaks | Treatment, follow-up | Patients, physician, facility staff | Synchronous | videoconference | Yes |
| Joshi et al. (2020) | USA | High income | Describe the screening algorithms for patients with SARS-CoV-2–related complaints and results of using a telehealth program to increase access to care. | Screening, diagnosis, follow-up | Patients, physician, clinical team (nurse, …) | Both | Phone call, patient portal | Yes |
| Guest et al. (2020) | USA | High income | Understand whether oropharyngeal swab (OPS), saliva, and dried blood spot (DBS) specimens collected by participants at home and mailed to a laboratory were sufficient for use in diagnostic and serology tests of SARS-CoV-2. | Diagnosis | Patients, physician | Both | Video call, email | No |
| Kim et al. (2020) | Korea | High income | Introduce the Korean Medicine (KM) telemedicine center for treatment of COVID patients in Korea | Treatment, follow-up | Patients, physician | Synchronous | Phone call | Yes |
| Strik et al. (2020) | USA | High income | Using apple watch for monitoring and avoiding arrhythmic cardiac complications | Follow-up | Patients, physician | Asynchronous | Mobile application (apple watch) | Yes |
| Rosen et al. (2020) | USA | High income | Use of a physical therapy tele rehabilitation program for inpatients with Covid-19 based on a specific algorithm | Treatment | Patients, physician, clinical team (nurse, …) | Synchronous | Videoconference | Yes |
| Soriano et al. (2020) | Spain | High income | Develop an application to help already overwhelmed hospital staff to actively monitor and assess COVID-19 infections and compatible symptoms in a population of hospital workers. | Screening | Hospital workers, health manager | Asynchronous | Mobile application | Yes |
| Zarghami et al. (2020) | Iran | Upper middle income | Utilizing tele-psychiatry to assess psychiatric comorbidities in COVID-19 patients and follow-up visits based on the patient’s condition. | Treatment, follow-up | Patients, physician | Synchronous | Mobile application | No |
| Sinha et al. (2020) | USA | High income | Describe the implementation and evaluation of a video visit program at a large, academic primary care practice in New York, the epicenter of the COVID-19 pandemic. | Triage, follow-up | Patients, physician, clinical team (nurse, …) | Synchronous | Mobile application | Yes |
| Sakai et al. (2020) | Japan | High income | Describe the effectiveness and risk management of remote rehabilitation for coronavirus disease (COVID-19) patients in general wards. | Treatment | Patients, physician, clinical team (nurse, …) | Synchronous | audio/video call | Yes |
| Vilendrer et al. (2020) | USA | High income | Describing key features and early learning of inpatient telemedicine programs in the hospital setting to reduce pathogen exposure, conserve personal protective equipment, and facilitate care personnel work. | Treatment, follow-up | Patients, physician, clinical team (nurse, …) | Synchronous | Mobile application, video call | Yes |
| Sivan et al. (2020) | UK | High income | Describe the development of an integrated rehabilitation pathway using telemedicine approach for individuals recovering from COVID-19 in the community | Follow-up | Patients, physician | Synchronous | Phone call | Yes |
| Tomlinson et al. (2020) | UK | High income | Introducing the use of telecommunications for routine ward round assessment. | Treatment, follow-up | Patients, physician | Synchronous | Phone call | Yes |
| Perlman et al. (2020) | Israel ,USA | High income | Describe the characteristics and symptoms of people who use digital health tools to address COVID-19–related concerns. | Screening | Patients, physician | Asynchronous | Mobile application | Yes |
| Ratwani et al. (2020) | USA | High income | Describe an on-demand telehealth approach for remote COVID-19 patient screening, patient characteristics, wait time, visit duration, technical success of the telehealth request and the post-visit trajectory of the patients. | Screening | Patients, physician, clinical team (nurse, …) | Synchronous | Phone call, video call | No |
| Li et al. (2020) | China | Upper middle income | Present the utilization of telemedicine technology in the specific context of the COVID-19 pandemic and postulate takeaways for global healthcare systems. | Screening, triage, treatment, follow-up | Patients, physician | Both | Mobile application, video call | Yes |
| Lian et al. (2020) | China | Upper middle income | Design and implementation of a web-based COVID-19 service platform at a tertiary hospital in China as well as the preliminary results of the implementation. | Screening, triage, treatment | Patients, physician | Both | Mobile application, website | Yes |
| García et al. (2020) | Spain | High income | Assess if telemedicine with telemonitoring is a clinically useful and safe tool for monitoring patients with COVID-19. | Follow-up | Patients, physician, clinical team (nurse, …) | Synchronous | Phone call | Yes |
| Lisker et al. (2020) | USA | High income | Describe a homecare based ambulatory protocol to evaluate, monitor, and treat moderate to high risk COVID-19 patients in their homes | Triage, treatment, follow-up | Patients, physician, clinical team (nurse, …) | Both | Phone call, email | Yes |
| Lopez-Villegas et al. (2020) | Spain | High income | Evaluate the effectiveness of implementation a teleconsultation Protocol in a Hospital Emergency Department | Triage, prevention | Patients, physician | Synchronous | Phone call, videoconference | No |
| Lwin et al. (2020) | Australia | High income | Describe a COVID Hospital-in-the-Home (HITH) service and clinical presentation of COVID-19 patients. | Screening | Patients, physician, clinical team (nurse, …) | Synchronous | Phone call, videoconference | No |
| Nivet et al. (2020) | France | High income | Evaluate the accuracy of diagnoses of COVID-19 based on chest CT as well as inter-observer agreement between tele radiologists during on-call duty and senior radiologists in suspected COVID-19 patients. | Diagnosis | Physician | Asynchronous | Tele radiology software | Yes |
| [Rabuñal et al. (2020)](https://www.liebertpub.com/doi/10.1089/tmj.2020.0144?url_ver=Z39.88-2003&rfr_id=ori:rid:crossref.org&rfr_dat=cr_pub%20%200pubmed) | Spain | High income | Using a telemedicine tool (TELEA) in the management of patients at risk, in a rural environment with a dispersed population in Lugo. | Follow-up | Patients, physician, clinical team (nurse, …) | Both | phone call, videoconference, email | No |
| [Nascimento et al. (2020)](https://journals.sagepub.com/action/doSearch?target=default&ContribAuthorStored=Nascimento%2C+Bruno+R) | Brazil | Upper middle income | Assess the impact of a large-scale COVID-19 telemedicine system on emergency department (ED) visits and all-cause and cardiovascular hospital admissions. | Triage, follow-up | Patients, physician, clinical team (nurse, …) | Both | Mobile application, phone call, video call | No |
| Reeves et al. (2020) | USA | High income | Design and implementation of EHR based rapid screening processes, laboratory testing, clinical decision support, reporting tools, and patient-facing technology related to COVID-19 | Screening, triage, treatment, follow-up | Patients, physician, clinical team (nurse, …) | Both | Mobile application, phone call, video call | Yes |
| Nunziata et al. (2020) | Italy | High income | Describing the specific pathway used to manage children with or exposed to COVID 19 infection based on the shared decision between experts using telemedicine tools. | Triage, diagnosis, treatment | Physician | Synchronous | Phone call | Yes |
| Reforma et al. (2020) | USA | High income | Describe the feasibility, clinical and process outcomes associated with a multidisciplinary telemedicine surveillance model to triage and manage obstetric patients with known exposures and/or symptoms concerning for COVID-19 | Screening, follow-up | Patients, physician, clinical team (nurse, …) | Synchronous | Phone call | No |
| Rigamonti et al. (2020) | Italy | High income | Report the experience on telemedicine conducted by hepatologists in a tertiary-care Center for Liver Disease of a University Hospital in Northern Italy for a 2-week period during the COVID-19 pandemic | Follow-up | Patients, physician | Both | Phone call, email | No |
| Martin et al. (2020) | UK | High income | Deploy the HoloLens2 MR device to support the delivery of remote care in COVID-19 hospital environments | Prevention | Physician | Synchronous | Mobile application, mixed-reality headset | No |
| Liu et al. (2020) | China | Upper middle income | Design, develop, and deploy a mobile-based decision support system for COVID-19 to assist GPs in collecting data, assessing risk, triaging, managing, and following up with patients during the COVID-19 outbreak. | Triage, treatment, follow-up | Patients, physician | Both | Mobile application | No |
| Lin et al. (2020) | Taiwan | High income | Evaluate the benefits and feasibility of a double triage and telemedicine protocol in improving infection control in the emergency department (ED) | Triage, prevention | Patients, physician, clinical team (nurse, …) | Synchronous | Video call | Yes |
| Dalla Costa et al. (2020) | Italy | High income | Explore the prevalence and impact of the current pandemic among MS patients across Europe using a remote monitoring system (RADAR-CNS). | Screening | Patients | Asynchronous | Smartwatch device and mobile sensors | No |
| Blazey-Martin et al. (2020) | USA | High income | Developing an algorithm, an EMR component, and a twice daily population report for managing COVID-19 patients remotely. | Triage, follow-up | Patients, physician, clinical team (nurse, …) | Both | Phone call, email | No |
| Cellai and O’Keefe (2020) | USA | High income | Study of identified patients with coronavirus disease 2019 in a telemedicine clinic who requested ongoing follow-up calls 6 weeks after symptom onset | Follow-up | Patients, physician | Synchronous | Phone call | No |
| Becker et al. (2020) | USA | High income | Describe the rapid implementation and sequential process improvement (PI) of a centralized tele hospitalist service to coordinate and optimize management of large number of COVID-19 patients in a tertiary and quaternary care hospital | Prevention, treatment | Patient logistics, hospitalist medicine, ipc, and patient experience teams | Synchronous | Phone call, videoconference | Yes |
| Barrett et al. (2020) | Ireland | High income | Describe an automated text-based active surveillance system which was used in Cork/ Kerry for the first 7 weeks of the COVID-19 response. | Prevention, follow-up | Patients, physician | Both | Phone call, text message | No |
| Schinköthe et al. (2020) | Germany | High income | Implement a free-of-charge, web- and app-based tool for patient assessment to assist health care professionals | Screening, treatment, follow-up | Patients, physician | Both | Mobile application, website, email | Yes |
| Zhang et al. (2020) | USA | High income | Development of a COVID-19 symptom screening application and report aggregate usage data from the first three months of its use across the organization. | Screening | Physician | Asynchronous | Mobile application | Yes |
| Zuccotti et al. (2020) | Italy | High income | Describes the Active Home Surveillance System (Operations Center for Discharged Patients; COD19) and the Home Hospital Care System (COD20) and presents the clinical data collected and the level of user satisfaction with the service | Treatment, follow-up | Patients, physician | Synchronous | Phone call, video call | No |
| Yamamoto et al. (2020) | Japan | High income | Develop a PHR-based COVID-19 symptom-tracking app and to demonstrate the practical use of health observations for COVID-19 using a smartphone or tablet app integrated with PHRs | Prevention | Patients, epidemiologists | Both | Mobile application, phone call | Yes |
| Sitammagari et al. (2020) | USA | High income | Describe the development and rapid deployment of a virtual hospital program, Atrium Health hospital at home (AH-HaH), within a large health care system | Treatment, follow-up | Patients, physician, clinical team (nurse, …) | Both | Mobile application, videoconference | Yes |
| Xu et al. (2020) | china | Upper middle income | Evaluate a telemedicine model that was developed to address the challenges of treating patients with progressive COVID-19 who are home-quarantined and shortages in the medical workforce. | Follow-up | Patients, physician | Both | Mobile application, email | Yes |
| Timmers et al. (2020) | Netherlands | High income | Assess people’s use of an app to support them with COVID-19 education, self-assessment, and monitoring of their own health for a 7-day period | Follow-up | Patients, physician | Asynchronous | Mobile application | Yes |
| Rodler et al. (2020) | Germany | High income | Assessing the effectiveness of the actions taken and the treatment modifications adapted for the patients with cancer at academic centers directly facing the challenges of the COVID-19 pandemic | Follow-up | Patients, physician | Both | Phone call, email | No |
| Gong1 et al. (2020) | China | Upper middle income | Explore the role of internet hospitals during the prevention and control of the COVID-19 | Screening, prevention, follow-up | Patients, physician | Synchronous | Mobile application | No |
| Chou et al. (2020) | USA | High income | Design a protocol to create a protective physical barrier with telemedicine technology to limit COVID-19 exposure in ED. | Prevention | Patients, physician | Synchronous | Videoconference | Yes |
| Kim et al. (2020) | Korea | High income | Development of a remote brief severity scoring system to assign priority for hospitalization and arranging for facility isolation. | Screening | Patients, physician | Synchronous | Phone call | Yes |
| Jones et al. (2020) | USA | High income | Transition inpatient diabetes management services to a virtual care model | Treatment | Patients, physician, clinical team (nurse, …) | Synchronous | Mobile application | Yes |
| Khairat et al. (2020) | USA | High income | Explore the trends in confirmed COVID-19 cases in North Carolina, and to understand patterns in virtual visits related to symptoms of COVID-19. | Screening, triage | Patients, physician | Synchronous | Phone call, video call | Yes |
| Jiang et al. (2020) | China | Upper middle income | Minimize psychological damages and provide timely assistance to the prevention and control of the epidemic | Treatment | Patients, physician | Synchronous | Phone call, video call | Yes |
| Yasaka et al. (2020) | USA | High income | Develop an effective contact tracing smartphone app that respects user privacy by not collecting location information or other personal data. | Prevention | Patients | Asynchronous | Mobile application | Yes |
| Wei et al. (2020) | China | Upper middle income | Introduce an internet-based integrated intervention to COVID-19 patients with psychological distress | Treatment | Patients | Asynchronous | Mobile application | No |
| Annis et al. (2020) | USA | High income | Propose a patient education and COVID-19 Remote Patient Monitoring (RPM) Solution | Follow-up | Patients, physician | Synchronous | Phone call, video call | No |
| Drew et al. (2020) | USA and UK | High income | Develop a COVID-19 Symptom Tracker mobile application to collect data for long-term studies as well as for immediate public health planning | Screening | Patients, physicians, epidemiologists, public authorities | Asynchronous | Mobile application | Yes |
| Mann et al. (2020) | USA | High income | Study the transformational impact of COVID-19 on telemedicine-driven health | Screening | Patients, physician | Synchronous | Videoconference | No |

**Supplementary Table 2: Outcomes and main findings of the included studies**

| **First author, Date** | **Outcome** | **Main findings** |
| --- | --- | --- |
| Heo et al. (2020) | Descriptive analysis of usage pattern include frequency of access and location of the user using google analytics | The application has been widely used in Korea as well as in other countries. It indicates the app's success and the community's need for it. In total, 83,460 users accessed the application 105,508 times. Interestingly, a significant number of older people have used the app. This could be due to the simple design of the app pages. |
| Lam et al. (2020) | Descriptive analysis of patients | The study has shown the feasibility of a virtual care program using a collaborative approach in the management of outpatients diagnosed with COVID-19. |
| Kouroubali et al. (2020) | Descriptive analysis of system development | The study described a platform and components that designed upon an existing personal health record to facilitate symptom tracking, self-management, and personalized recommendations, effective communication channels between patients and clinicians and public health authorities. |
| Jethwa et al. (2020) | Descriptive analysis of patients, personal protective equipment (PPE) use | PPE usage including masks, gowns, and gloves in the first week of CVC implementation reduce dramatically. |
| Ford et al. (2020) | Descriptive analysis of patients, personal protective equipment (PPE) use | There are substantial benefits in utilizing telehealth during the COVID-19, including the ability to rapidly scale the number of patients being screened and providing continuity of care and the ability to monitor and track patients through linking telehealth systems to the EHR. Also there was a significant reduction in healthcare worker exposure and time spent donning and doffing PPE, and conservation of a substantial amount of PPE. |
| Krenitsky et al. (2020) | Descriptive analysis of televisit, no show rate | The outcome of the majority of telehealth visits was to continue outpatient management, with only 3.9% of visits requiring escalation of care to inpatient evaluation. The no show rate for the virtual COVID-19 clinic visits was 13.4%. |
| Heslin et al. (2020) | Descriptive analysis of system development and duration of using the telehealth program | The telehealth program was used for 880 minutes of call time and 523 encounters in 30 days. Emergency department telehealth program decreased exposure to COVID-19 and conserved personal protective equipment. |
| Kassaye et al. (2020) | Descriptive analysis of patients | The symptom tracker system was designed based on guiding principles developed during peer consultations. 48 Georgetown University School of Medicine students or their social contacts entered data into the system. One of the 48 users (2%) reported active COVID-19 infection and had no symptoms by the end of the monitoring period. |
| Judson et al. (2020) | Time to visit scheduling, sensitivity and specificity of detecting emergency-level care, descriptive analysis of accessing and completing the self-triage and self-scheduling tool | The patients who directly scheduled visits had a significantly shorter time from starting the self-triage and self-scheduling tool to scheduling a visit compared to those who called the hotline to schedule. The sensitivity and specificity of detecting emergency-level illness was 87.5% and 76.2%, respectively. |
| Harris et al. (2020) | Hospitalization rate, mortality rate | The hospitalization and mortality rates were 37.5% and 12.5%, respectively, which were significantly lower than documented in other facility outbreaks. |
| Joshi et al. (2020) | Visit volume, number of SARS-CoV-2 tests, number of positive tests | There was a greater than seven-fold increase in telehealth patient volume. 33% were recommended for SARS-CoV-2 testing. 20% had a positive result. It can increase access to community-based care, decrease clinician exposure, and minimize the demand for personal protective equipment. |
| Guest et al. (2020) | Biological sufficiency of the participant-collected oropharyngeal swab (OPS), saliva, and dried blood spot (DBS) specimens | Most participant-collected OPS (96.7%), saliva (96.1%), and DBS (93.1%) specimens are suitable and sufficient for testing for SARS-CoV-2 RNA and serology as judged by clinical observers. Data support the utility of participant-collected and mailed-in specimens for SARS-CoV-2 testing. |
| Kim et al. (2020) | Descriptive analysis of patients consulted and prescriptions | Telemedicine center provided treatments to an average of 192 patients per day. The number of treatments per patient is distributed from 1 to over10 times. Also, 4,552 herbal medicines were prescribed through the telemedicine center. |
| Strik et al. (2020) | Inter observer variability (ICC), agreement between cardiologists on QTc measurement accuracy (Cohen’s kappa (κ)) | There was excellent agreement between cardiologists on whether recordings were acceptable for QTc measurements (κ=0.92). Inter observer agreement for QTc measurements was high with an ICC of 0.92. |
| Rosen et al. (2020) | Descriptive analysis of patients received telerehabilitation, in-person rehabilitation, or a combination of the two | No adverse events occurred to patients receiving telerehabilitation PT only; therefore, the telerehabilitation therapists were not required to enter patient rooms. The use of inpatient telerehabilitation reduced staff exposure while providing essential education and services to patients. |
| Soriano et al. (2020) | Descriptive analysis of hospital staff participant, response rate | It was observed a decline in adherence after an initial participation peak in some hospitals. Other sites were characterized by low participation rates (2%-6%) throughout the study period. |
| Zarghami et al. (2020) | Descriptive analysis of patients and mental health status | Psychiatric disorders were significantly more common in patients with (60%) than those without (28.8%) hospital admission and more frequent in female versus male subjects. |
| Sinha et al. (2020) | Descriptive analysis of patients, system effectiveness, user’s satisfaction | Of 1,475 video visits were scheduled, 69.8% were completed. 2.0% failed due to technical problems and were converted to telephone visits. Patients reported high satisfaction with their video visit (mean score of 4.6 on a 5-point scale). |
| Sakai et al. (2020) | Destination after discharge, PCR results, mobility scores on level surfaces, and Barthel Index total scores | Early remote rehabilitation was performed in 32.6% of cases. Finally, 41.9% of the patients received remote rehabilitation. Remote rehabilitation minimizes contact with COVID-19 patients in terms of frequency of staff entering the red zone and their duration of stay in the red zone, which could prevent transmission of infection and reduce the amount of PPE needed. |
| Vilendrer et al. (2020) | Descriptive analysis of video visits and feasibility report | Positive adoption with 631 inpatient video calls lasting an average of 16.5 minutes based on inclusion criteria. Rapid deployment of inpatient telemedicine is feasible in diverse settings as a response to the COVID-19 pandemic. |
| Sivan et al. (2020) | Describe rehabilitation pathway and referral thresholds | A pragmatic, simple, comprehensive and integrated rehabilitation pathway was developed to screen COVID-19 survivors and target specific rehabilitation interventions in a timely and efficient manner, based on the previously designed screening tool. |
| Tomlinson et al. (2020) | exposure time, user’s satisfaction | The results show a significant reduction in face-to-face exposure time in patients receiving teleconsultations. This was associated with positive patient feedback and high satisfaction levels, with several patients identifying the importance of telecommunications. |
| Perlman et al. (2020) | Descriptive analysis of patients | Individuals were relatively young and predominantly female. Users who chose to communicate with a remote physician were more likely to have been classified as requiring immediate medical evaluation by the COVID-19 self-assessment. |
| Ratwani et al. (2020) | Waiting time, visit duration | 61.6% of on-demand telehealth requests had a visit reason that was likely COVID-19 related. The average completed encounter wait time was 26.5 min and the mean visit length was 8.8 min, providing convenient, low-barrier access to providers. |
| Li et al. (2020) | Descriptive analysis of visits | 10,557 online COVID-19 consultations were conducted for 6,662 individuals. Through this analysis, it is apparent that telemedicine technologies are a legitimate mechanism for screening, triaging and treating patients with COVID-19 |
| Lian et al. (2020) | Descriptive analysis of patients and visits | 96,642 people had used the automated COVID-19 screening and symptom monitoring systems 161,884 and 7,795,194 times, respectively. According to the volume of provided services, it seems the digital solutions of automated COVID-19 screening, daily symptom monitoring, web-based care, and knowledge access have commendable acceptability and feasibility for complementing offline hospital services and facilitating disease control and prevention. |
| García et al. (2020) | Descriptive analysis of patients and visits | The data from the study suggested that at-home monitoring with telemedicine and telemonitoring in patients with COVID-19 is a well-accepted, useful, and safe system. |
| Lisker et al. (2020) | Descriptive analysis of patients and visits | A total of 182 patients were enrolled during the study. There were no unexpected admissions or deaths. The CROWN program has demonstrated the feasibility and apparent safety of a specialized, Home-Care based protocol for the ambulatory management of moderate to high risk COVID-19 patients. |
| Lopez-Villegas et al. (2020) | Descriptive analysis of patients and visits, personal protective equipment (PPE) costs | More than 98% of the cases managed and discharged with the system were able to be monitored without the need for a second examination. Also, it allowed the doctors to discharge a large number of patients, without the need for direct contact or the use of PPEs (45% of the cases resolved). |
| Lwin et al. (2020) | Descriptive analysis of patients and visits | Approximately half of the patients only required telehealth assessment. Emotional support by the HITH COVID team has been highly appreciated and valued by patients and their family during home isolation. |
| Nivet et al. (2020) | Diagnostic accuracy based on chest CT | The study found that inter-observer agreement was excellent between on-call radiologists with varying degrees of experience and senior radiologists. |
| [Rabuñal et al. (2020)](https://www.liebertpub.com/doi/10.1089/tmj.2020.0144?url_ver=Z39.88-2003&rfr_id=ori:rid:crossref.org&rfr_dat=cr_pub%20%200pubmed) | Descriptive analysis of patients and visits | With the appropriate selection, a subgroup of high-risk COVID-19 patients can be managed by telemedicine, which is likely to avoid emergency room visits and hospital admissions. |
| [Nascimento et al. (2020)](https://journals.sagepub.com/action/doSearch?target=default&ContribAuthorStored=Nascimento%2C+Bruno+R) | Descriptive analysis of patients and visits and system usage | In 60 days, 24,354 patients accessed one of the telemedicine systems. The system resulted in low rates of ED visits and hospital admissions, suggesting positive impacts on healthcare utilization. Cardiovascular admissions were remarkably rare. |
| Reeves et al. (2020) | Description of system development | The study described a series of EHR enhancements designed to support the rapid deployment of new policies, procedures, and protocols across a healthcare system in response to the COVID-19 pandemic. |
| Nunziata et al. (2020) | Descriptive analysis of patients and visits, system usage and reasons for teleconsultations | Application of stringent criteria for hospital admission based on clinical conditions, risk factors and respect of biocontainment measures, allowed to manage the majority of cases (71.1%) through telemedicine. A telephone call lasted 13 minutes on average and generally led to a shared decision between the experts within the Hub Centre and the pediatricians. |
| Reforma et al. (2020) | Descriptive analysis of patients and visits | The majority of patients (86%) were appropriately managed in the outpatient setting and did not require in-person evaluation. Patients were enrolled in the telemedicine model for a median of 7 days and averaged one phone call daily. |
| Rigamonti et al. (2020) | Descriptive analysis of patients | The study emphasized the usefulness of telemedicine for maintaining the continuity of care among patients with autoimmune liver diseases during the pandemic. |
| Martin et al. (2020) | Acceptability, feasibility, exposure time, personal protective equipment (PPE) use | The deployment of the HoloLens2 led to a 51.5% reduction in time exposed to harm for staff and an 83.1% reduction in the amount of PPE used. The majority of staff using the device agreed it was easy to set up and comfortable to wear, improved the quality of care and decision making, and led to better teamwork and communication. |
| Liu et al. (2020) | Accuracy of risk stratification | The average value of the three classification results of the macro–area under the curve were all above 0.71 in a different scenario. |
| Lin et al. (2020) | exposure time, patient's evaluation time | The total exposure time in the telemedicine group was 4.6 minutes shorter than that in the conventional group and the total evaluation time in the telemedicine group was 2.8 minutes longer than that in the conventional group. |
| Dalla Costa et al. (2020) | Descriptive analysis of patients | 399 patients of RADAR-MS have been included with 87/399 patients (21.8%) reporting major symptoms suggestive of COVID-19. A trend for an increased risk of COVID-19 symptoms under alemtuzumab and cladribine treatments in comparison to injectables was observed. |
| Blazey-Martin et al. (2020) | Description of system development | Population management strategy helped to optimize at-home care for our COVID-19 patients and enabled to identify those who require inpatient medical care in a timely fashion. |
| Cellai and O’Keefe (2020) | Descriptive analysis of patients | For a subset of patients with COVID-19 (4.7% in our cohort), symptom duration is more than 5 weeks and impacts their ability to return to work and activity. |
| Becker et al. (2020) | Description of system development | Telehospitalist service line can be set up and adapted to settings without an existing telemedicine infrastructure. It is essential to establish well thought-out consensus workflows that all members of the multidisciplinary team get repeatedly educated and updated on Campbell. |
| Barrett et al. (2020) | Descriptive analysis of patients | The text message-based system resulted in the detection of additional positive cases and helped to break chains of transmission in the community. |
| Schinköthe et al. (2020) | Description of system development | The described system can support the care by real-time, electronic communication between a patient and their physician, including telehealth, remote patient monitoring, and secure communication between clinicians and their patients. |
| Zhang et al. (2020) | Description of system development and participants | The screening application was being used by over 25,000 employees each weekday. After three months, 2,169,406 attestations were recorded with COVID Pass. Over this period, 1865/160,159 employees (1.2%) reported positive symptoms. Also, 1,865 symptomatic employees were identified who otherwise may have come to work, potentially putting others at risk. |
| Zuccotti et al. (2020) | Description of system development and participants, user’s satisfaction | The service took charge of 1097 patients and conducted a total of 27,195 calls. Overall, the service was well accepted by the patients. Only five patients gave at least one negative rating of the relationship with the care provision, ease of measuring the clinical parameters or level of general satisfaction with the service. Negative ratings came from asymptomatic patients. |
| Yamamoto et al. (2020) | Description of system development and system usage | The app greatly reduced the follow-up burden of individuals who had close contact with known cases of confirmed COVID-19 infection. It was discovered 72 health observers had close contact with a confirmed case. Among them, 57 (79.2%) adopted the use of the health observation app and 14 used telephone investigations. |
| Sitammagari et al. (2020) | Descriptive analysis of participants, length of stay, intensive care unit (ICU) admission, mechanical ventilation, mortality | None of the patients in the VACU received noninvasive ventilation. None died during their hospital admission. Among those 40 transferred patients, 16 required ICU admission, 7 required mechanical ventilation, and 2 died during their hospital admission. |
| Xu et al. (2020) | Description of system development and system usage, descriptive analysis of patients | Among 188 individuals using the telemedicine system, 114 (60.6%) were not infected with COVID-19 and were dismissed. Of the 74 confirmed patients with COVID-19, 26 (35%) recovered during the study period and voluntarily stopped using the system. The remaining 48/76 confirmed patients with COVID-19 (63%) used the system until the end of the study, including 6 patients whose conditions progressed to severe or critical. |
| Timmers et al. (2020) | System usage, user’s satisfaction | A total of 6194 people used the app. Over 5000 users used the self-assessment tool and over 1300 users started the symptom diary. Furthermore, the app received positive evaluations related to the satisfaction with the information and the added value of keeping track of symptoms. |
| Rodler et al. (2020) | Descriptive analysis of patients | Virtual management and reductions in the frequency of visits are feasible and confine the infection risk for patients with uro-oncological cancer after the COVID-19 pandemic. |
| Gong1 et al. (2020) | Descriptive analysis of patients | A total of 4913 consultations were analyzed. The counselees’ motivation and the doctors’ recommendation for offline visits were inconsistent, indicating improper medical-seeking behaviors. |
| Chou et al. (2020) | Description of system development | A telemedicine strategy has been developed and broadly implemented in many EDs. The protocol using onsite telemedicine has become a safer way for both physicians and nurses. |
| Kim et al. (2020) | Waiting time | The brief severity scoring system for COVID-19 worked safely to decline waiting patients and solve the hospital-bed shortage. |
| Jones et al. (2020) | Glycemic outcomes | Transitioning to virtual care models does not limit the glycemic outcomes of inpatient diabetes care and should be employed to reduce patient and provider exposure in the setting of COVID-19. |
| Khairat et al. (2020) | Descriptive analysis of patients and trends in confirmed covid-19 transmission | The most significant spread of the disease occurs in areas with a high population density and in areas with significant airports. Virtual care can provide efficient triaging in the counties with the highest number of COVID-19 cases. |
| Jiang et al. (2020) | Description of system development | A psychological crisis intervention (PCI) has been initiated via remote medical services to minimize the risk of infections among psychiatrists and psychologists, and to minimize psychological damages. |
| Yasaka et al. (2020) | Description of system development | The proposed smartphone-based contact tracing method presents a novel solution that preserves privacy while demonstrating the potential to suppress an epidemic or pandemic outbreak. |
| Wei et al. (2020) | Psychological distress | The proposed internet-based integrated intervention showed a rapid improvement on mental health, and reducing levels of depression and anxiety in COVID-19 patients. |
| Annis et al. (2020) | user’s satisfaction | Rapid and successful deployment of a remote monitoring and caring system for COVID-19 patients, which helped them stay safe at home with great satisfaction. There were 300 patients responded to the patient satisfaction questions within the application, of which 74% answered that they would be extremely likely to recommend their doctor, 18% were slightly likely, 5% were neutral, 0.7% were slightly unlikely, and 2% were extremely unlikely resulting in a Net Promoter Score of 66.5% |
| Drew et al. (2020) | Data on risk factors, predictive symptoms, clinical outcomes, and geographical hotspots | Based on the analyses with a larger sample set of COVID-19 symptoms, anosmia appears to be a strong predictor for COVID-19. A weighted prediction model was developed based on the symptoms of more than 2 million individual app users. |
| Mann et al. (2020) | Descriptive analysis of patients and visits, user’s satisfaction | Telemedicine urgent care volume grew from 82 visits to 1,336 after 15 days. Of these visits, 55.3% were COVID-19 related, outpacing the 381 COVID-19 visits in all the NYULH emergency rooms that day. The proposed video-based visit promotes social distancing with high patient satisfaction levels. |
